# How useful are post consultation letters to patients?

**DOI:** 10.1186/1741-7015-4-2

**Published:** 2006-01-20

**Authors:** Nicola J Roberts, Martyn R Partridge

**Affiliations:** 1 NHLI Division, Imperial College London, Respiratory Health Services Research Group, Charing Cross Hospital, St Dunstans Road, London, W6 8RP, UK

## Abstract

**Background:**

As part of the NHS plan it was suggested that all patients receive copies of letters sent to their General Practitioner following outpatient consultations. The former Secretary of State for Health extended this proposal, suggesting that patients have a specific letter to themselves after a hospital consultation.

**Methods:**

The aim of this study was to send cardiorespiratory patients attending Charing Cross Hospital, a copy of the letter sent to their G.P. plus a specific letter to themselves and to assess the usefulness and comprehensibility of each. The letters were analysed for dictation time, Flesch Reading Ease Score, Flesch-Kincaid Grade Level and word count. Eighty-four out of 105 sequential patients (80%) consented and were sent both types of letter after their attendance. Patients returned both letters circling any items they did not understand and stated a preference for the GP letter, patient letter, or both. The patients' GPs were subsequently also asked for their views on each letter.

**Results:**

GP letters took significantly longer to dictate than patient letters. The Flesch Reading Ease Score was significantly higher in the patient letters, indicating that the patient letters were easier to read. The GP letters were significantly longer than the patient letters and patients were significantly more likely to circle more items in the GP letters (p < 0.001). The content of letters is sometimes inaccurate. Thirty-six out of 62 patients (58%) would like to receive both letters, 13/62 (21.6%) would prefer the GP letter and 13/62 (20%) wanted only the patient letter. 45 GPs replied (62.5%), 28/45 (62.5%) wanted the GP letter, 14 GPs (31.1%) wanted both letters and 3/45 (6.7%) wanted the patient letter only. General themes concerned insufficient clinical details and the GPs preferred the structure of the letters written to them.

**Conclusion:**

Patients appreciate copies of the letter being sent to their GP but comprehension is less good than with a shorter letter written especially to the patient. More attention needs to be paid to making letters to GPs simpler to read without losing the structure and detail liked by GPs. A compromise might be to dictate the letter in front of the patient and to provide a speciality-specific glossary to accompany each letter.

## Background

As part of the NHS plan it was suggested that all patients should have the opportunity to receive copies of the letters sent to their General Practitioner following outpatient consultations [1]. The former Secretary of State for Health extended this proposal, suggesting that patients should have a specific letter to themselves after a hospital consultation [2].

Sending copies of GPs' letters to patients has been the practice of some doctors for many years and has been shown to be associated with increased patient satisfaction [3]. There is less agreement regarding the proportion of patients reporting that they understand the content [3,4] but nearly every previous study has reported that patients who have received letters wished to continue to do so [5,6]. We wished to understand better the logistics and possible benefits of a specially dictated letter to the patient compared with a copy of a letter being sent to their GP.

## Methods

This project received ethical approval from the Riverside Research ethics committee. Consultants were asked to dictate the two letters in alternating order for each patient and recorded on a stopwatch the time taken for dictating each letter. Consultants adopted usual practice and wrote to the general practitioners in the way they usually would. The letters were analysed for dictation time, Flesch Reading Ease Score, Flesch-Kincaid Grade Level [7,8] and word count.

Both letters were sent to the patients by post within a few days of their consultation and the patients were asked to:

a. circle any words, terms or phrases which they could not understand;

b. express a preference for which letter they would wish to receive.

General practitioners (GPs) of all of the patients involved in the study were sent a questionnaire asking which letter they preferred and which letter they would like to receive if it was normal practice, and to list up to 3 deficiencies if only the patient letter was sent to them. They were also invited to make any other comments. Themes were developed for the deficiencies reported by the GPs in the patient letter.

Quantitative analysis was carried out using the statistical package SPSS version 11 and the Wilcoxin Signed Ranks test.

## Results

One hundred and five (new and follow-up) patients attending Cardiology and Respiratory outpatient clinics at Charing Cross Hospital were invited to take part in the study. Eighty-four patients consented (80%) and were sent a letter especially dictated for them and a copy of the letter written by the hospital consultant to their GP. Out of 84 patients, 62 (72.6%) completed both the questionnaire and returned the two letters; 2 returned the letters only.

### 1. Format, content and comprehensibility of letters

No instructions were given to the consultants but all used headings at the beginning of the letter (to the GP) summarising the case, and most included a paragraph on tests performed and treatment advised. Despite not receiving any advice as to how to write the letters to patients, all consultants spontaneously adopted a rather discursive chatty letter style whereas to GPs the letters were more traditionally structured with headings and lists. The letters to the general practitioners took significantly longer to dictate than the letters to the patients. The Flesch Reading Ease Score was significantly higher (i.e. easier to read) for the patient letters than for the GP letters (Table 1). The mean Flesch-Kincaid Grade level score for the letters written to GPs was 10.72 ± 1.43, and for the letters written to patients was 11.04 ± 1.39. GP letters were significantly longer (444 ± 169.87 words) than the patient letters (351.5 ± 129.05 words, p < 0.001) and patients were significantly more likely to circle more items in the GP letters (p < 0.001). Forty-seven respondents (74.6%) circled no items and 16/63 (25.4%) circled between 1 and 5 items in the patient letters. On the GP letters, 32 respondents (50.8%) circled no items but 31/63 (49.2%) circled 1–12 items. Respondents preferred the patient letter to the GP letter when asked their preferences (45.2% versus 33.8%). When asked which letter they would prefer to receive, 36/62 patients (58%) said they would like to receive both letters, 13/62 (21.6%) would prefer the GP letter and 13/62 (20%) wanted only the patient letter.

### 2. Patients' responses to the two types of letters

The letters written to the patients had a total of 32 circled items; GP letter contained 90 circled items. These were split into two categories: (a) terminology and (b) queries about the correctness of the content of an item (for example patient disagreeing with the statement "*you were not short of breath"*). In the patient letters there were 22 queries about terms and 10 queries about the factual content (for example, date of onset of symptoms). In comparison, in the GP letters 76 terms were circled and there were 14 queries regarding the content. Some examples of medical or technical terms about which the patients were unclear are listed in Table 2.

### 3. General practitioners' responses to the patient letter

A short questionnaire was sent to 72 general practitioners (GPs) each of whom had one or more patients who had participated in this research study. Forty-five GPs responded (62.5%). GPs were asked which letter they preferred and, if such letter writing was normal practice, which letters would they like to receive. Out of the 45, 40 (88.8%) preferred the letter written to the GP. When asked which letter/s they would like to receive, 28/45 (62.5%) wanted the GP letter, 14/45 (31.1%) wanted both letters, and 3/45 (6.7%) wanted the patient letter only.

Each GP was asked to list the three most important deficiencies if only the patient letter was sent to them (Table 3).

When asked for additional comments, GPs said they did not like the chatty nature of the patient letters; several commented on the extra workload and time involved to read and summarise extra letters or less structured letters. Five GPs suggested that patients should get an improved GP letter and that patients could go to the GP with any queries.

## Discussion

Fifty-eight percent of patients wanted copies of both letters suggesting that no single letter was adequate, and they specifically wanted to see what their GP was being told. In contrast, GPs preferred the GP letter for the layout, content of clinical information and advice on treatment and management. The time and cost of sending duplicate letters is a practical issue raised by the GPs, and their suggestion to improve the GP letter and send a single letter to both patient and GP is a practical solution. There are obviously imperfections in the GP letters with a less satisfactory Reading Ease Score and more items circled as unclear. One solution might be to provide patients with a list of terms possibly relevant to their illness in addition to their letter. Whilst the Flesch Reading Ease score suggested that the letters written specifically to patients were easier to read, all the letters to Cardiorespiratory patients required a high level of understanding with a grade 10/11 level reading age. The average reading level in the US is estimated to be approximately 8^th ^grade [9]. The general reading age of the UK population is estimated at about 9 years [10]. Factual inaccuracies were present in both patient and GP letters according to the patients, and this problem may be reduced if the letters are dictated in front of the patient at the end of the consultation [11].

## Conclusion

Patients appreciate receiving copies of the letter being sent to their GP but comprehension is less good than with a shorter letter written especially to the patient. However, for neither the majority of patients nor the GPs would the specific patient letter be sufficient and more attention needs to be paid to making GP letters simpler to read without losing the structure and detail liked by GPs. The content of letters to GPs is sometimes incorrect and this may be remedied by dictating the letter in front of the patient. A compromise that would satisfy both the GP and patients is a structured letter to the GP copied to the patients but amended to meet patients' needs using simpler language and a glossary. However, occasional letters specifically to patients may be indicated in complex situations.

### What is already known about this?

*Patient recall during consultations is poor *[12].

*Most patients want copies of letters from outpatients clinics and find them useful *[5].

### What this study has found

*Comprehension of a letter written specifically to patients is higher when compared to the copy of the GP letter*.

*Both types of letters frequently contain both factual inaccuracies and terminology that is not understood*.

### Recommendations

• Further attention needs to be given to writing more comprehensible letters to GPs.

• A speciality-specific glossary may need to be given to patients to aid understanding.

• Dictating letters in front of patients [11] provides an opportunity for patients to correct factual errors.

## Competing interests

MRP and NJR do not have any significant competing interests (financial or non-financial).

## Authors' contributions

MRP developed the study design and protocol. NJR was responsible for implementing the study and analysing the results. Both contributed to drafting the report and approved the final manuscript.

## Pre-publication history

The pre-publication history for this paper can be accessed here:



## Figures and Tables

**Table 1:** Summary of GP and patient letters (n = 84)

**Type of post consultation letter**	**Letter to General Practitioner**	**Letter to patient**	**Significance** ** (Wilcoxin Signed Ranks test)**
**Length of time to dictate letter (minutes ± SD, n)**	3.28 ± 2.2, 81	2.57 ± 1.42, 82	p = 0.019
**Flesch Reading Ease Score**	49.76 ± 9.1, 84	55.44 ± 9.26, 84	p < 0.001
**Flesch-Kincaid Grade Level**	10.72 ± 1.43	11.04 ± 1.39	p = 0.062
**Circled items in each letter**	31/63 circled 1–12 items	16/63 circled 1–5 items	p < 0.001
**Word Count**	444 ± 169.87	351.5 ± 129.05	p < 0.001

**Table 2:** Items circled by patients in both letters

**Terms in cardiology letters**	**Terms in respiratory letters**
Heart sounds 1+2	Apnoea syndrome
GTN	Nocturia
Myocardial	Polysomnography
Aortic	Pulmonary
Infarction – peak troponin	Bronchiectasis
Mitral regurgitation	Daytime somnolence
Cardiac syncope	Oropharyngeal isthmus
Atrial fibrillation	Haemoptysis
CCS class II	Nasal CPAP
0.7 peak gradient, 85 mean gradient 48 ejection fraction	FEV_1_/FVC
OD	RCP score
Q waves	Spirometry

**Table 3:** GPs' listed deficiencies in patient letter

	**Number of responses**
Lack of terminology/ missing results and investigations	46
General detail and style of letter	23
Diagnosis/Drug therapy (missing/inadequate)	16
Other – (e.g. GP less involved)	8
Medical summary (missing/inadequate)	6
Patient history (missing/inadequate)	2
Impairs GP-Consultant relationship	2
